# Prevalence and sequence analysis of equid herpesviruses from the respiratory tract of Polish horses

**DOI:** 10.1186/s12985-018-1018-3

**Published:** 2018-07-11

**Authors:** Karol Stasiak, Magdalena Dunowska, Jerzy Rola

**Affiliations:** 1 Department of Virology, National Veterinary Research Institute, Al. Partyzantow 57, 24-100, Pulawy, Poland; 2 Institute of Veterinary, Animal and Biomedical Sciences, Massey University, Palmerston North, New Zealand

**Keywords:** Equine herpesvirus, EHV-2, EHV-5, Phylogeny, Quantitative PCR, Virological survey

## Abstract

**Background:**

Equid herpesviruses (EHVs) are widespread in equine populations worldwide. While the infection with equine α-herpesviruses (EHV-1 and EHV-4) has been linked to several clinical outcomes, the pathogenic potential for equine γ-herpesviruses (EHV-2 and EHV-5) is still unclear. The objective of the current study was to determine the prevalence of infection with EHVs among Polish horses, to investigate factors associated with EHV infections among horses sampled, and to determine genetic variability within Polish EHV-2 isolates.

**Methods:**

Virus-specific real-time PCR assays were used for detection of EHV-1, EHV-2, EHV-4 and EHV-5 in nasal swabs collected from 540 horses from 13 national horse studs located throughout Poland. A proportion of EHV-2/5 positive samples were subjected to virus isolation followed by amplification and analysis of partial glycoprotein B sequence.

**Results:**

Overall, 448/540 (83.0%) horses sampled were positive for at least one virus. The most prevalent was infection with EHV-2 (77.2%), followed by EHV-5 (47.0%), and EHV-4 (0.4%). None of the horses was positive for EHV-1. Approximately half of the virus-infected horses were positive for both EHV-2 and EHV-5. The proportion of EHV-2/5 positive horses varied by age, breed, and season. Only 8.0% of horses sampled, mostly Arabians, showed clinical signs of respiratory disease at the time of sampling. The viral load of both EHV-2 and EHV-5 DNA was highest in swabs from young horses, which was particularly evident for EHV-2 infected foals. Mean viral loads in nasal swabs collected from diseased horses were higher than in swabs from healthy horses. That was also true for EHV-2 when only diseased Arabian foals were considered, but the levels of EHV-5 DNA were lower in swabs from diseased than from healthy foals. In agreement with other studies, there was a considerable variability between Polish EHV-2 sequences, with no clustering of sequences from horses with different health status. The level of EHV-2 variability seemed to differ between different studs/breeds.

**Conclusions:**

The presence of foals and yearlings on a property is likely to increase the risk of active EHV-2/5 infection among in-contact horses. The existence of breed-specific differences in susceptibility to EHV-2/5 infections should be further investigated, as it may provide one variable that needs to be considered in attempts to associate EHV-2/5 infections with disease. Overall, the data presented add to the existing knowledge of the epidemiology and biology of equine γ-herpesviruses, with the long-term goal of better understanding of the pathogenesis and the impact of infections with these viruses on the well-being of the horse.

## Background

Horses are natural hosts to at least five equid herpesviruses (EHVs) including equine α-herpesviruses EHV-1 and EHV-4, and equine γ-herpesviruses EHV-2 and EHV-5 [1]. Due to high economic impact, EHV-1 remains particularly important, as infection with this virus can result in various disease outcomes including upper respiratory tract disease, abortion, neonatal foal death, or neurological disease, termed equine herpesvirus myeloencephalopathy [2–4]. Infection with a closely related EHV-4 has been typically linked to upper respiratory disease only [5, 6].

The disease association for γ-herpesviruses EHV-2/5 has been difficult to establish [7, 8], as both viruses have been identified in samples from clinically normal horses, as well as from horses with respiratory disease [9–12]. It has been suggested that EHV-2/5 infections may be linked to poor performance in horses, with or without overt disease [13–15]. This was based on increased frequency of detection of EHV-2/5 from tracheal washes of horses affected by airway inflammation, although results of other studies failed to demonstrate such a relationship [16, 17]. In addition to respiratory disease, EHV-2/5 infections have been linked to keratoconjunctivitis [18, 19], although both viruses have also been detected from conjunctival swabs of clinically normal horses [20, 21]. Recently, EHV-2 and EHV-5 have also been detected in the gastric mucosa, which raised a question of their possible role in the development of gastric ulcers [22]. Equid herpesvirus 5 has been also implicated as an etiological agent for fibrotic lung disease in adult horses termed equine multinodular pulmonary fibrosis [23].

The controversy over EHV-2/5 involvement in equine respiratory disease likely reflects, at least in part, differences between various study designs including the type of samples collected, timing of sampling, definition of respiratory disease used, or the age-structure of the populations sampled. It is also possible that EHV-2/5 infections, even if subclinical on their own, predispose to infections with other pathogens. If so, clinical disease observed would depend on what other pathogens circulate among populations sampled. This seems to be supported by the fact that experimental vaccination with an iscom-based subunit vaccine against EHV-2 protected foals against a life-threatening pneumonia due to *Rhodococcus equi* infection [24]. Finally, discrepancies in the conclusions reached by various authors with regard to EHV-2/5 disease association may be due to the possible existence of viruses with different biological properties. This has been suggested based on marked genomic heterogeneity observed for both EHV-2 and EHV-5 [25–27].

Genomes of EHV-2 and EHV-5 consist of genes conserved between different herpesviruses that are interspersed with species-specific genes and non-coding regions [28]. The conserved herpesviral genes include that coding for glycoprotein B (gB). This protein is essential for virus replication and plays a role in virus entry into the cells [29]. The gB of both EHV-2 and EHV-5 is a dilsulphate-linked heterodimer that forms an integral part of the viral envelope [30, 31]. The variability in amino acid sequence between different EHV-2 gB sequences has been mapped to three main regions (sites I, II and III) using monoclonal antibodies, with the viruses examined forming two main antigenic groups: EHV-2.86/67-like and EHV-2.141-like based on variability at site I [32]. This site is located in the N-terminus of EHV-2/5 gB exposed on the surface of the virions and contains neutralizing epitopes that are important targets for the immune response [31, 32]. The highest degree of variability between various EHV-2/5 viruses was mapped to site III. This hypervariable site is located between amino acids 415 and 448 in EHV-2.86/67 gB sequence (NP_042604.1), immediately N-terminal to the conserved endoproteolytic cleavage site [31, 32]. In a subsequent study [33] three main phylogenetic lineages of EHV-2 (groups 1, 2, and 3) were described based on phylogenetic analysis of a region (aa 235–609 of EHV-2 gB and 234–605 of EHV-5 gB) that included hypervariable site III of 18 field isolates of EHV-2/5.

Equine herpesviruses have worldwide distribution including Poland [6, 34]. However, EHV-1 detection in Poland has been reported mainly as part of diagnostic investigations of cases of abortion or neonatal death [35–38] and no data are currently available on the frequency of detection of EHV-1/4 among other groups of horses. The only virological survey of EHV-2 among Polish horses was conducted approximately 15 years ago [39]. Information on EHV-5 infections among Polish horses is limited to one case report published in a national journal [40], and hence not easily accessible to non-Polish speaking readers.

Hence, the objective of the current study was to determine prevalence of infection with equine herpesviruses among Polish horses, to investigate factors associated with herpesvirus infections among horses sampled, and to determine genetic variability within Polish EHV-2 isolates.

## Methods

### Horses

A total of 540 horses from 13 national horse studs were included in the study (Table 1). Nasal swabs (standard Sigma Virocult® swab - 15 cm long with a cellular foam bud) were collected from each horse on a single visit to the stud between April 2015 and May 2016 by one of the authors (KS) except for studs II and III. At each stud, samples were collected from all mares and foals available on the visit day. In addition, swabs were collected from youngsters, with an aim to sample approximately 10 yearlings and 10 two-year-olds at each stud. The health status of horses was recorded at the time of sampling. Horses were considered to be affected by respiratory disease if they showed nasal discharge or coughing. Samples from horses at studs II and III were provided by attending veterinarians as part of diagnostic investigation of outbreaks of respiratory disease at those studs. Each swab was placed into 2 mL of the universal transport medium (Sigma Virocult®, Medical Wire and Equipment, Ltd) at the time of sampling and transported to the Department of Virology of the National Veterinary Research Institute in Pulawy (Poland) on ice packs within 24–48 h from collection.

**Table 1:** Description of horses (*n* = 540) sampled on one occasion between April 2015 and May 2016 at each of the 13 Polish stud farms included in a virological survey of equine herpesviruses

Stud farm	Breed^a^	Horses sampled [*n*]	Median Age (IQR) [years]	Respiratory disease [*n*]	Region	Sampling date
I	Arabian	64	1 (0.5–5.5)	5	Lubelskie	Jun 2015
II	Arabian	19	0.5 (0.5–7.0)	19	Swietokrzyskie	Apr 2015
III	Arabian	18	1 (1.0–1.0)	18	Lubelskie	Feb 2016
IV	Hucul horse	42	1 (0.5–2.0)	0	Malopolskie	Mar 2016
V	Malopolska horses	42	1 (1.0–2.0)	0	Opolskie	Apr 2016
VI	Polish Halfbred horse	37	0.5 (0.5–12.0)	0	Opolskie	Apr 2016
VII	Wielkopolska horse	47	1 (0.5–4.0)	0	Wielkopolskie	May 2016
VIII	Wielkopolska horse	48	3 (2.0–9.0)	0	Wielkopolskie	May 2016
IX	Wielkopolska horse	30	2 (0.5–6.2)	0	Warminsko-mazurskie	Apr 2016
X	Silesian horse	44	4 (0.5–10.0)	0	Dolnoslaskie	Apr 2016
XI	Thoroughbred	60	2 (0.5–9.7)	1	Mazowieckie	Apr 2016
XII	Polish Coldblood horse	49	1 (1.0–2.0)	0	Kujawsko-pomorskie	Apr 2016
XIII	Polish Konik	40	1.5 (0.6–3.5)	0	Warminsko-mazurskie	Jun 2015
Total		540		43		

^a^For the description of Polish breeds see http://pzhk.pl/en/breeding/polish-breeds/

### Processing of samples

Upon arrival at the laboratory, each swab was vortexed in situ for 30 s to release viruses into the transport medium and discarded. An aliquot (20–30 μL) of each transport medium was used to create pooled samples from seven to 12 animals from the same stud and age group. All samples were stored at − 80 °C. Total DNA was extracted from both pooled and individual samples using the QIAamp DNA Mini Kit (Qiagen, Hilden, Germany), according to the manufacturer’s instructions.

### Real-time PCR assays

Quantitative PCR (qPCR) assay targeting conserved regions of gB genes of EHV-1 and EHV-4 [41] was used in the study. Each qPCR reaction (25 μL) consisted of forward and reverse primers (Table 2) at a final concentration of 200 nM each, 100 nM of EHV-1 probe, 400 nM of EHV-4 probe, and 2 μL of template DNA in 1× TaqMan® Universal PCR Master Mix (Life Technologies). The following cycling conditions were used: uracyl-DNA glycosylase treatment at 50 °C for 2 min, initial denaturation at 95 °C for 10 min, followed by 40 cycles at 95 °C for 15 s and 55 °C for 60 s. Negative (water) and positive (EHV-1 438/77 and EHV-4 405/76, ATCC) controls were included in each run. Quantitative PCR assays for EHV-2 and EHV-5 were conducted with primers and probes targeting gB gene (Table 2), as described by Hue et al. [42]. Each virus-specific PCR reaction consisted of forward and reverse primers at a final concentration of 400 nM each, 200 nM of the probe and 2 μL of template DNA in 1× TaqMan® Universal PCR Master Mix (Life Technologies). The PCR included uracyl-DNA glycosylase treatment at 50 °C for 2 min, initial denaturation at 95 °C for 10 min, followed by 45 cycles at 94 °C for 10 s and 52 °C for 30 s. Negative (water) and positive (EHV-2 VR-701, ATCC and EHV-5 DNA extracted from a field virus, confirmed by sequencing) controls were included in each run. All reactions were performed using a StepOne Plus™ Real-Time PCR System.

**Table 2:** Primers and probes used for detection of equid herpesvirus 2 (EHV-2) and EHV-5 among horses in Poland

Assay	Virus	Region	Primers / probes (5′ to 3′)	Size (bp)	Reference
Real-time PCR	EHV-2	gB	Forward: GTGGCCAGCGGGGTGTTC	78	[42]
			Reverse: CCCCCAAAGGGATTYTTGAA		
			Probe: FAM-CCCTCTTTGGGAGCATAGTCTCGGGG-TAMRA		
	EHV-5		Forward: AACCCGCCGTGCATCA	66	
			Reverse: AGGCGCCACACACCCTAA		
			Probe: FAM-ACAACACCACCAACCCCTTTCTGCTG-TAMRA		
Conventional PCR	EHV-2		Forward: GATGGTCTCACCTCTAGCAT	1111	[12]
			Reverse: CTGGTGTAACACAGGTCTTC		
	EHV-5		Forward: CCAACACAGAAGACAAGGAG	1339	
			Reverse: CACGGTGATACAGTCAGAGA		

Fluorescent probes were dually labelled with carboxyfluorescein (FAM) and tetramethylrhodamine (TAMRA)

For the EHV-1 and EHV-4 qPCR Cq values below 38.0 were considered positive, and Cq values below 41.0 and 37.0 were considered positive for EHV-2 and EHV-5 assays, respectively. The EHV-1/4 qPCR was applied as a qualitative assay (positive/negative). In order to construct a standard curve for quantification of EHV-2 and EHV-5 DNA, serial dilutions of a stock solution containing either recombinant plasmid DNA (EHV-2, 10^6^ to 10^0^ copies/μL) or gel-purified 518 bp PCR product containing the sequence targeted by qPCR (EHV-5, 9.0 × 10^7^ to 9.0 × 10^0^ copies/μL) were used. To transform Cq values obtained in qPCR assays to number of viral copies, we applied the formula: Amount = 10^((Cq-b)/m)^, where m is the slope and b is the intercept from the regression equation. DNA from the pooled samples was used as a template in the first round of qPCR assays. The assays were repeated with individual samples from positive pools. All samples from negative pools were considered negative for a given virus.

### Virus isolation

Virus isolation was aimed at EHV-2 and EHV-5 only. It was performed using all samples collected from diseased horses as well as selected samples from healthy horses. The latter generally included samples with relatively low Cq values (below 26.0) in either EHV-2 or EHV-5 qPCR. A portion of nasal swab transport medium (350 μL) was supplemented with 25 μL of Antibiotic Antimycotic Solution (Sigma-Aldrich), filtered through 0.45 μm membrane syringe filter (La-Pha-Pack) and inoculated into one well of a 24-well tissue culture plate (Nunc) containing 90% confluent rabbit kidney (RK-13) cells. The cells were propagated in Eagle’s Minimum Essential Medium (Sigma-Aldrich) supplemented with 10% fetal bovine serum and 1% Antibiotic Antimycotic Solution (Sigma-Aldrich). The plates were incubated at 37 °C in a humidified 5% CO_2_ atmosphere for seven days and checked every day for the presence of a viral cytopathic effect (CPE). Cultures showing CPE were frozen and the lysates tested for the presence of EHV-2/5 using virus specific qPCR. Cultures were considered negative for virus growth if no CPE was present after a total of three blind passages. During each passage, cultures were freeze-thawed and 200 μL of cell culture lysate was inoculated onto fresh RK-13 cells as described above.

### Sequence analysis

Viral DNA from 67 EHV-2 positive cultures, two EHV-5 positive cultures and seven EHV-2 positive swabs collected from healthy Arabians (stud I) was used as a template in conventional PCR assays targeting gB gene (Table 2), with the expected products of 1111 bp for EHV-2 and 1339 bp for EHV-5, as described by Wang et al. [12]. Each PCR reaction consisted of 0.2 mM deoxynucleotide mix (Sigma-Aldrich), 0.5 μL JumpStart AccuTaq LA DNA Polymerase (Sigma-Aldrich), 600 nM of each primer and 2 μL of template DNA in a supplied 1× buffer in a total volume of 25 μL. Amplifications were performed in a Biometra Thermocycler (Biometra, Germany) using the following cycling conditions: 5 min of initial denaturation at 94 °C, followed by 35 cycles of denaturation (1 min at 94 °C), annealing (for EHV-2: 1 min at 61.7 °C / for EHV-5: 1 min at 59.5 °C), extension (1 min at 72 °C) and final extension (7 min at 72 °C). PCR products were visualized following electrophoresis through a 1.5% ethidium bromide stained agarose gel for 30 min at 90 V. Negative non-template controls were included in each PCR run.

PCR reactions containing products of the expected sizes were enzymatically purified (ExoSAP-IT PCR Product Cleanup Reagent, Thermo Fisher Scientific) and the products were commercially sequenced using BigDye® Terminator version 3.1 (Applied Biosystems) at the Genomed (Warsaw, Poland). The obtained sequences were assembled using BioEdit software (version 7.2.5) and trimmed to identical length (924 bp for EHV-2 and 1152 bp for EHV-5). Alignments were carried out using ClustalW in MEGA5. Phylogenetic trees were constructed using the maximum likelihood method (ML) with 1000 bootstrap value using the Kimura 2-parameter model in MEGA5 software [43].

### Statistical analysis

The relationships between categorical variables (age, stud farm, breed, season, disease status) and the presence of viral infection was investigated using contingency tables. Age was categorized into: foals (< 1 year-old), yearlings, horses 2 to 5-year-old, and in 5-year intervals for horses older than 5 years. Fisher’s exact test was used to analyse 2 × 2 tables, and Pearson’s Chi-square test to analyze tables with more than two variables. To investigate which horses had an increased risk of infection with a specific virus within each main category, each group was compared to the remaining population enrolled into the study, and the *p* values were adjusted using Bonferroni correction. Descriptive statistic was used to summarize measurement variables. For comparisons of viral loads between horses from different groups the qPCR data were transformed to log values. Comparisons between two groups were performed using two-tailed unpaired t test if the variances for the groups were not significantly different. Welch correction was applied to the comparisons if variances differed significantly between the groups. For comparisons of more than two groups, Kruskal-Wallis test with Dunn’s comparison was used. For all statistical analysis, the significance level was set at *p* <  0.05. All analyses were performed using GraphPad Prism version 5.04 (GraphPad Software, San Diego California USA, www.graphpad.com).

## Results

### Infection with equid herpesviruses

**Fig. 1:**
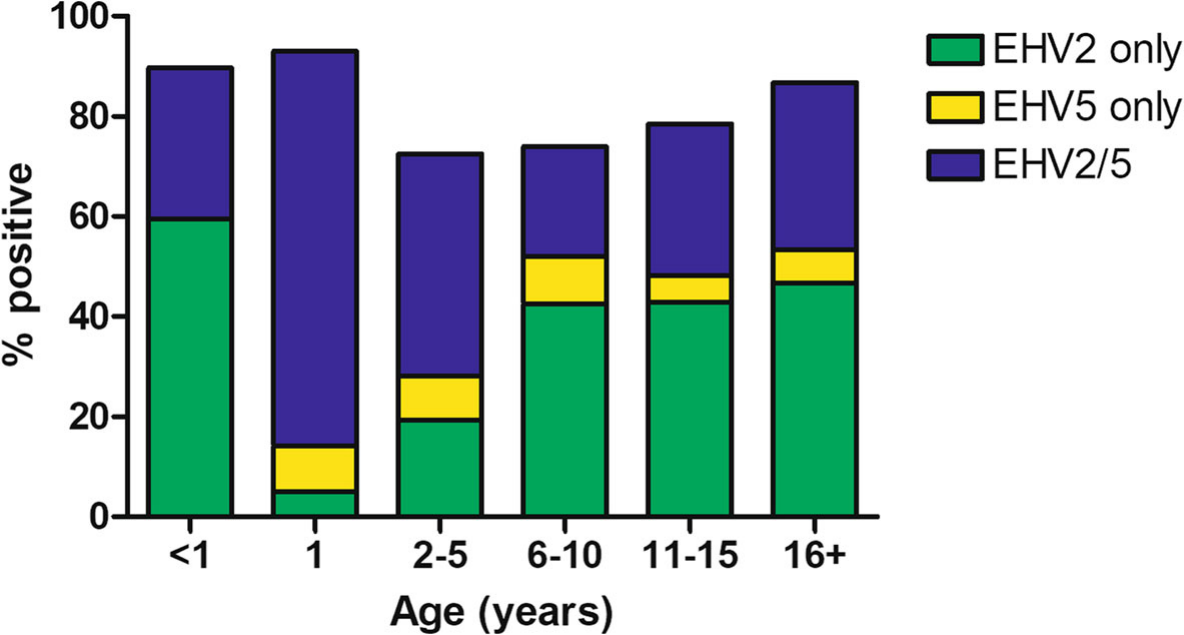
Frequency of detection of equid herpesvirus-2 (EHV-2) and EHV-5 in nasal swabs from horses included in the study (*n* = 540) based on virus-specific quantitative PCR

Overall, 448/540 (83.0%) of horses sampled were positive for at least one virus (Fig. 1). The most prevalent was infection with EHV-2 (417/540, 77.2%), followed by EHV-5 (254/540, 47.0%), and EHV-4 (2/540, 0.4%). None of the horses was positive for EHV-1 (Table 3). Approximately half (222/448, 49.6%) of the virus-infected horses were positive for both EHV-2 and EHV-5, with the remaining horses testing positive for EHV-2 only (193/448, 43.1%), EHV-5 only (31/448, 6.9%), EHV-2 and EHV-4 (1/448, 0.2%) or EHV-2, EHV-5 and EHV-4 (1/448, 0.2%).

**Table 3:** Frequency of PCR detection of equid herpesvirus 2 (EHV-2) and EHV-5 in nasal swabs collected from Polish horses (*n* = 540) stratified by age, stud farm, breed, season at the time of sampling, and disease status

Category	Horses [*n*]	EHV-2			EHV-5		
		*n* (%)	RR (95% CI)	*p*	*n* (%)	RR (95% CI, p)	*p*
Age (years)							
< 1	173	155 (89.6)	1.3 (1.2–1.4)	< 0.0001*	52 (30.1)	0.6 (0.4 to 0.7)	< 0.0001*
1	99	83 (83.8)	1.1 (1.0–1.2)	0.0862	87 (87.9)	2.3 (2.0–2.7)	< 0.0001*
2–3	101	66 (65.4)	0.8 (0.7–1.0)	0.0024*	57 (56.4)	1.3 (1.0–1.5)	0.0462
4–9	83	52 (62.7)	0.8 (0.7–0.9)	0.001*	29 (34.9)	0.7 (0.5–1.0)	0.0171
10–15	69	49 (71.0)	0.9 (0.8–1.1)	0.2182	23 (33.3)	0.7 (0.5–1.0)	0.0196
16+	15	12 (80.0)	1.0 (0.8–1.3)	1	6 (40.0)	0.9 (0.5–1.6)	0.6119
Stud farm							
I	64	43 (67.2)	0.9 (0.7–1.0)	0.0556	46 (71.9)	1.6 (1.4–2.0)	< 0.0001*
II	19	17 (89.5)	1.2 (1.0–1.4)	0.2693	8 (42.1)	0.9 (0.5–1.5)	0.8159
III	18	18 (100.0)	1.3 (1.3–1.4)	0.018	16 (88.9)	2.0 (1.6–2.4)	0.0004*
IV	42	31 (73.8)	1.0 (0.8–1.2)	0.5686	26 (61.9)	1.4 (1.1–1.8)	0.0532
V	42	39 (92.9)	1.2 (1.1–1.4)	0.0115	29 (69.1)	1.5 (1.2–1.9)	0.0035*
VI	37	34 (91.9)	1.2 (1.1–1.3)	0.0254	13 (35.1)	0.7 (0.5–1.1)	0.1718
VII	47	41 (87.2)	1.1 (1.0–1.3)	0.1016	27 (57.5)	1.3 (1.0–1.6)	0.1683
VIII	48	36 (75.0)	1.0 (0.8–1.2)	0.7191	13 (27.1)	0.6 (0.3–0.9)	0.0038
IX	30	27 (90.0)	1.2 (1.0–1.3)	0.1151	11 (36.7)	0.8 (0.5–1.2)	0.264
X	44	24 (54.6)	0.7 (0.5–0.9)	0.0005*	4 (9.1)	0.2 (0.1–0.5)	< 0.0001*
XI	60	54 (90.0)	1.2 (1.1–1.3)	0.0134	27 (45.0)	1.0 (0.7–1.3)	0.7847
XII	49	41 (83.7)	1.1 (1.0–1.3)	0.2896	27 (55.1)	1.2 (0.9–1.6)	0.2934
XIII	40	12 (30.0)	0.4 (0.2–0.6)	< 0.0001*	7 (17.5)	0.4 (0.2–0.7)	0.0001*
Breed							
Arabian	101	78 (77.2)	1.0 (0.9–1.1)	1	70 (69.3)	1.7 (1.4–2.0)	< 0.0001*
Coldblood	49	41 (83.7)	1.1 (1.0–1.3)	0.2896	27 (55.1)	1.2 (0.9–1.6)	0.2934
Hucul	42	31 (73.8)	1.0 (0.8–1.2)	0.5686	26 (61.9)	1.4 (1.1–1.8)	0.0532
Malopolska horse	42	39 (92.9)	1.2 (1.1–1.4)	0.0115	29 (69.1)	1.5 (1.2–1.9)	0.0035*
Polish Halfbred	37	34 (91.9)	1.2 (1.1–1.3)	0.0254	13 (35.1)	0.7 (0.5–1.2)	0.1718
Polish Konik	40	12 (30.0)	0.4 (0.2–0.6)	< 0.0001*	7 (17.5)	0.4 (0.2–0.7)	0.0001*
Silesian horse	44	24 (54.6)	0.7 (0.5–0.9)	0.0005*	4 (9.1)	0.2 (0.1–0.5)	< 0.0001*
Thoroughbred	60	54 (90.0)	1.2 (1.1–1.3)	0.0134	27 (45.0)	1.0 (0.7–1.3)	0.7847
Wielkopolska horse	125	104 (83.2)	1.1 (1.0–1.2)	0.088	51 (40.8)	0.8 (0.7–1.1)	0.1254
Season							
Spring	186	121 (82.3)	1.4 (1.2 to 1.6)	< 0.0001*	185 (44.3)	0.8 (0.7 to 0.9)	0.0179
Summer	104	55 (52.9)	0.6 (0.5 to 0.8)	< 0.0001*	53 (51.0)	1.1 (0.9 to 1.4)	0.3837
Winter	18	18 (100.0)	1.3 (1.3 to 1.4)	0.018	16 (88.9)	2.0 (1.6 to 2.4)	0.0004
Respiratory disease							
Yes	43	41 (95.4)	1.3 (1.2–1.4)	0.0019*	26 (60.5)	1.3 (1.0–1.7)	0.0796
No	497	376 (75.7)			228 (45.9)		
Total	540	417 (77.2)			254 (47.0)		

Associations between categorical variables included in the analysis and infection with EHV-2 or EHV-5 were tested using contingency tables. Fisher’s exact test was used to analyse 2 × 2 tables, and Pearson’s Chi-square test to analyze tables with more than two variables. To calculate relative risk (RR) for EHV-2/5 infection within each main category, horses from each subcategory were compared with the remaining population enrolled into the study, and the *p* values were adjusted using Bonferroni correction

*Results were considered statistically significant for the following Bonferroni-adjusted *p* values: Age (< 0.0083), Stud farm (< 0.0038), Breed (< 0.0055), Season (< 0.017)

The proportion of horses positive for γ-herpesviruses EHV-2/5 varied for different age groups (Table 3). Foals were more likely (*p* <  0.0001) to be positive for EHV-2, less likely (*p* <  0.0001) to be positive for EHV-5 than older horses. Out of all age groups, yearlings were most likely (*p* <  0.0001) to be positive for EHV-5. The proportion of EHV-2/5 positive horses also varied by breed and season (Table 3). Horses sampled in spring were more likely to be shedding EHV-2 than horses sampled at other times of the year. Arabian horses and Malopolska horses were more likely to be positive for EHV-5 than horses from other breeds, while Silesian and Polish Konik horses were less likely to be positive for either EHV-2 or EHV-5 than horses from other breeds. The associations between breed and the likelihood of EHV-2/5 infection were generally reflected at the stud-farm level, with horses positive for EHV-2/5 being over-represented at two Arabian studs (studs I and III) and Malopolska horse stud (stud V), while under-represented at studs breeding Silesian horses (stud X) and Polish Konik horses (stud XIII).

### Respiratory disease

Overall, 43/540 (8.0%) horses sampled showed clinical signs of respiratory disease at the time of sampling. All but one of the diseased horses were Arabians, with 37/43 horses residing at studs II and III (Table 1). Unfortunately, samples from healthy horses were not available from studs II and III, and thus the association between EHV-2/5 infection and respiratory disease on these two studs could not be investigated.

### Viral DNA load

**Fig. 2:**
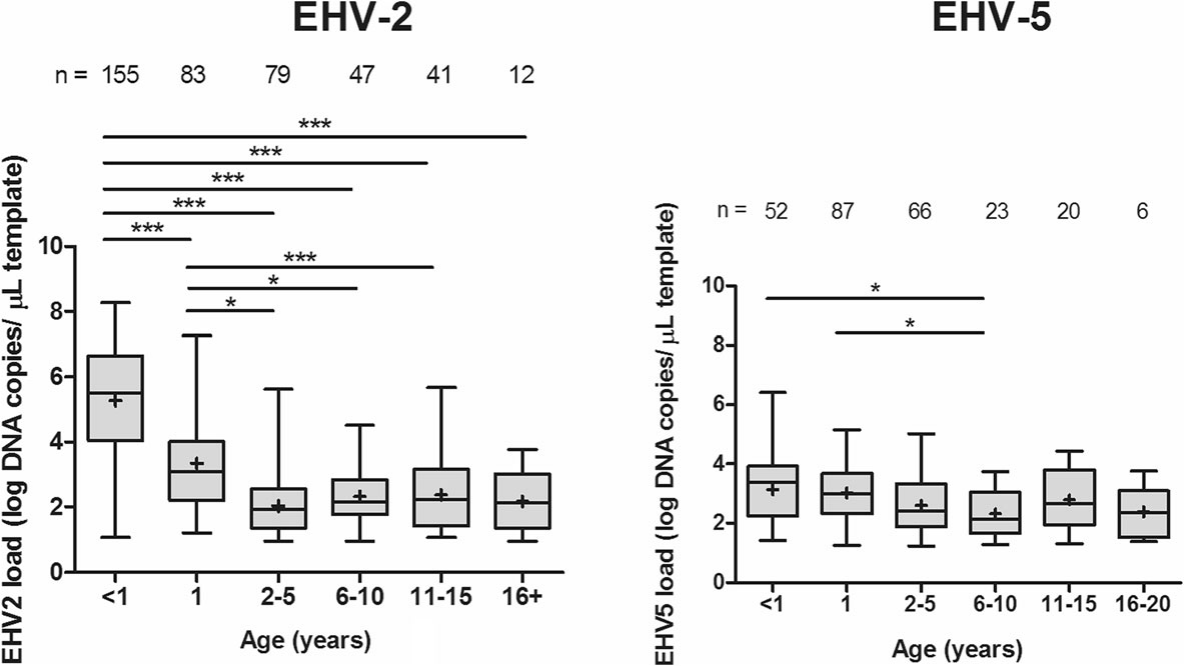
Viral DNA load of equid herpesvirus-2 (EHV-2) and EHV-5 in nasal swab samples collected from horses included in the study (*n* = 540) stratified by age. The middle line in each box represents median, whiskers represent minimum and maximum values, with means indicated by ‘+’. Significance levels calculated using Kruskal-Wallis test with Dunn’s multiple comparison are indicated by the stars: < 0.05 (*),< 0.001 (***). The number of horses in each category is indicated above each box

Mean viral load in nasal secretions was higher for EHV-2 (3.6 ± 1.9 log copies/μL) than for EHV-5 (2.8 ± 1.0 log copies/μL, *p* <  0.0001) when all EHV-2/5 positive horses were considered. Viral loads in nasal swabs from foals and yearlings were higher than viral loads in swabs from older horses (*p* <  0.001, Fig. 2), with the highest median viral DNA load detected in nasal secretions from EHV-2 infected foals (5.5 log copies/μL, IQR = 4.0–6.6), followed by EHV-2 infected yearlings (3.1 log copies/μL, IQR = 2.2–4.0). By comparison, the median load of EHV-5 DNA was 3.4 log (IQR = 2.2–3.9) copies/μL for foals and 3.0 log (IQR = 2.3–3.8) copies/μL for yearlings. The lowest median viral load was detected in nasal swabs from horses 2–5 years old for EHV-2 (1.9 log copies/μL, IQR = 1.3–2.6) and from horses 6–10 years old for EHV-5 (2.1 log copies/μL, IQR = 1.7–3.0).

There appeared to be differences between median loads of EHV-2 DNA between horses from different breeds (Kruskal-Wallis test, *p* = 0.0002), with Arabian horses showing the highest median load of EHV-2 (4.4 log copies/μL, IQR = 2.8–5.7). These differences were, however, not consistent when horses of similar ages were compared, with the highest median EHV-2 loads detected in nasal swabs from Polish Halfbred foals (6.6 log copies/μL, IQR = 5.7–6.9, *n* = 78), Arabian yearlings (4.5 log copies/μL, IQR = 3.5–5.9, *n* = 22), and adult (2-year-olds and older) Thoroughbreds (2.6 log copies/μL, IQR = 2.2–3.4, *n* = 26). Median loads of viral DNA did not differ significantly for horses from different breeds for EHV-5.

**Fig. 3:**
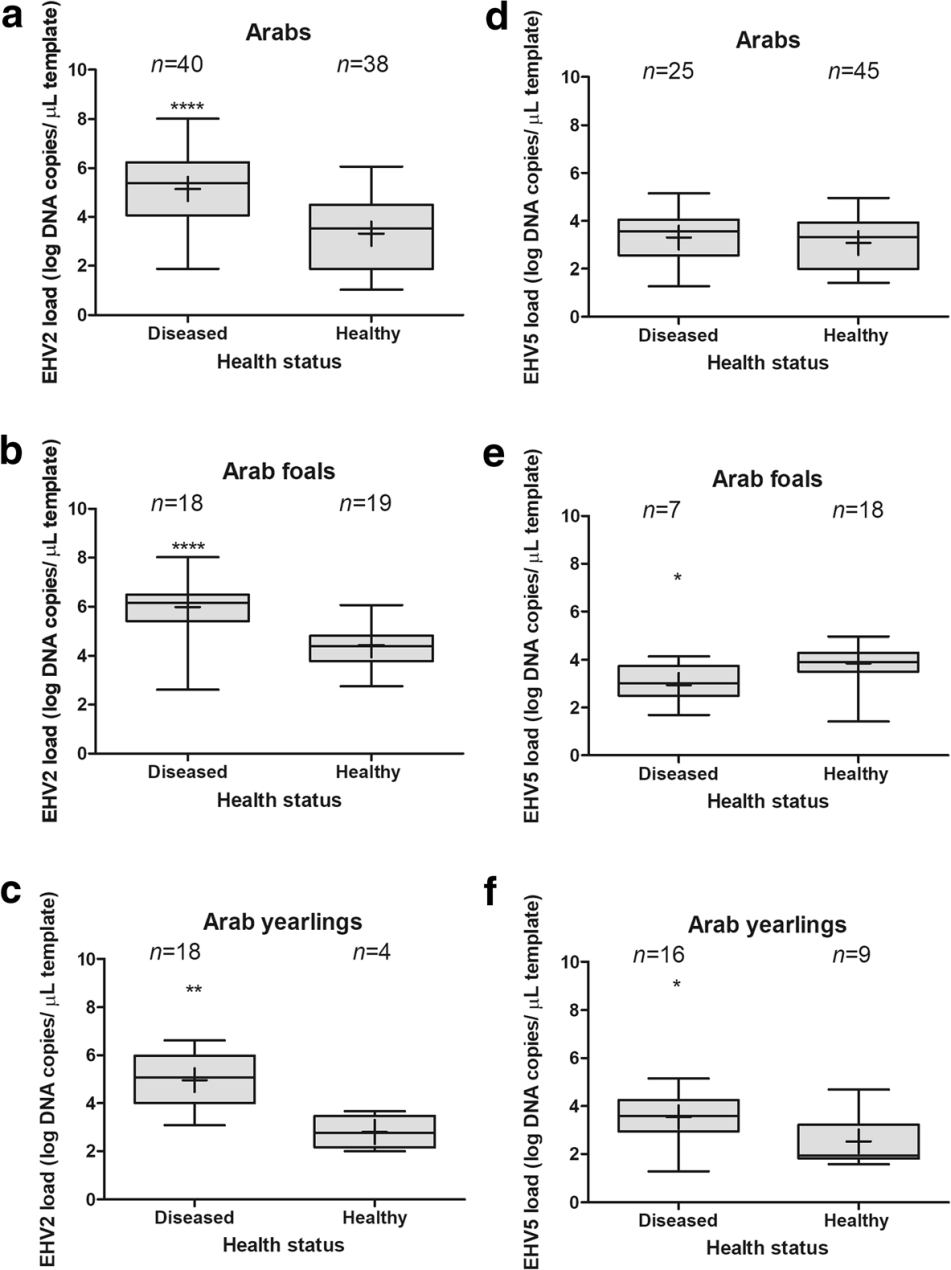
Viral DNA load of equid herpesvirus-2 (EHV-2) and EHV-5 in swabs from all Arabian horses tested (*n* = 101, **a** and **d**), Arabian foals (*n* = 39, **b** and **e**) and Arabian yearlings (*n* = 28, **c** and **f**), stratified by disease status. The middle line in each box represents median, whiskers represent minimum and maximum values, with means indicated by ‘+’. The number of virus-positive horses in each category is shown above each box

Mean viral loads in nasal swabs collected from diseased horses were higher than in swabs from healthy horses for both EHV-2 (5.2 ± 1.5 log copies/μL, *n* = 41 versus 3.4 ± 1.9 log copies/μL, *n* = 376, *p* <  0.0001) and EHV-5 (3.3 ± 1.0 log copies/μL, *n* = 26 versus 2.8 ± 1.0 log copies/μL, *n* = 228, *p* = 0.0204). This was also true for EHV-2 when only Arabian horses were included in the analysis (5.1 ± 1.6 log copies/μL versus 3.3 ± 1.5 log copies/μL, *p* <  0.0001) (Fig. 3a), and when the analysis was restricted to Arabian foals (6.0 ± 1.2 log copies/μL versus 4.4 ± 0.8 log copies/μL, *p* <  0.0001) (Fig. 3b) or Arabian yearlings (5.0 ± 1.1 log copies/μL versus 2.3 ± 0.7 log copies/μL, *p* = 0.0016) (Fig. 3c). In contrast, there was no difference between mean EHV-5 DNA load between diseased and healthy Arabian horses (3.3 ± 1.0 log copies/μL versus 3.1 ± 1.1 log copies/μL, *p* = 0.37) when all Arabian horses were considered (Fig. 3d). However, swabs from diseased Arabian foals showed lower EHV-5 DNA load than swabs from healthy Arabian foals (2.9 ± 0.8 log copies/μL versus 3.9 ± 0.8 log copies/μL, *p* = 0.0174, Fig. 3e), while swabs from diseased Arabian yearlings showed higher load of EHV-5 DNA than swabs from healthy Arabian yearlings (3.5 ± 0.9 log copies/μL versus 2.5 ± 1.0 log copies/μL, *p* = 0.0212, Fig. 3f).

### Virus isolation

Out of 146 samples subjected to virus isolation, 97 (66.4%) were positive for at least one EHV, including those positive for EHV-2 only (*n* = 67), EHV-5 only (*n* = 2), and both EHV-2 and EHV-5 (*n* = 28). Equid herpesvirus 2 was isolated from 95/143 (66.4%) samples positive for EHV-2 by PCR, with three samples testing negative by both virus isolation and PCR (kappa = 0.075, 95% SI -0.006 to 0.157). Equid herpesvirus 5 was isolated from 30/63 (47.6%) samples positive for EHV-5 by PCR, with 83 samples testing negative by both PCR and virus isolation (kappa = 0.508, 95% SI 0.380 to 0.637).

### Sequence analysis

Long PCR products were amplified from 50/67 EHV-2 qPCR positive cultures, 2/2 EHV-5 qPCR positive cultures and 7/7 EHV-2 qPCR positive swabs tested. Sequence alignments of partial gB gene from 57 EHV-2 Polish viruses showed 82.4 to 100% identity at the nucleotide level and 80.6 to 100% identity at the amino acid level. As expected, the highest degree of variability was observed N terminal to the conserved endoproteolytic cleavage site, which had the consensus amino-acid sequence (K/R)-(K/R)-R-R (aa 427 to 430 in NP_042604). The degree of identity between Polish EHV-2 viruses and foreign sequences was lower and ranged from 68.1 to 100% at the nucleotide level and from 63.6 to 100% at the amino acid level. The highest genetic variability was observed between Polish EHV-2 sequences from stud IX (80.6 to 100% at the amino acid level, *n* = 6). Viruses from studs III (*n* = 2), X (*n* = 3) and XIII (*n* = 4) were 100% identical to each other. Nearly all (11/12) EHV-2 sequences from stud I were closely related with 99.8 to 100% identity at the nucleotide level, irrespective of whether they were obtained from diseased (*n* = 5) or healthy (*n* = 6) horses. Identity between two Polish EHV-5 sequences obtained from horses from stud IV was 93.8 and 91.6% at the nucleotide and amino acid levels, respectively. Polish EHV-5 were 90.8 to 99.6% and 88.5 to 100% identical to EHV-5 sequences from other countries at the nucleotide and amino acid level, respectively.

**Fig. 4:**
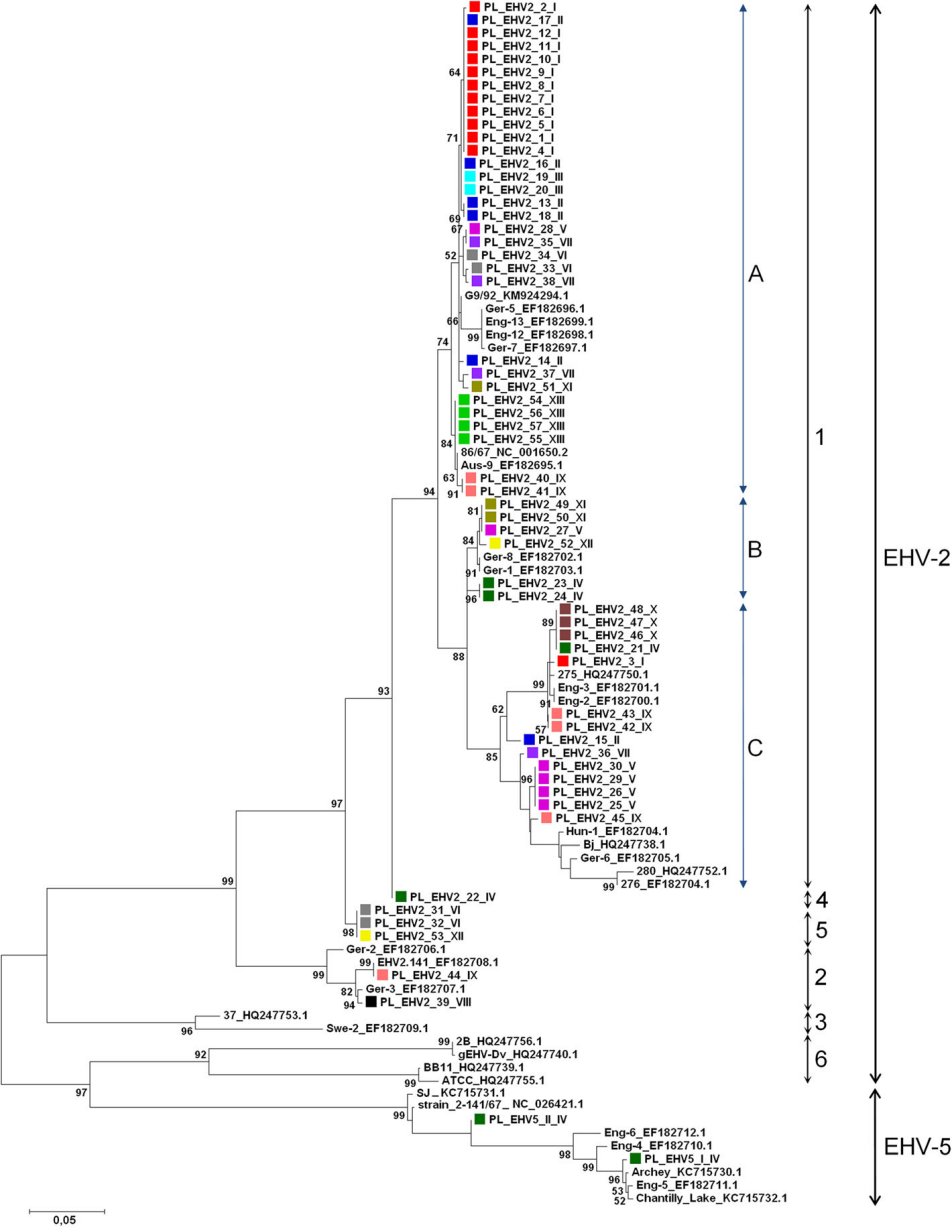
Phylogenetic tree of equid γ-herpesviruses (EHV) based on 726 bp fragment from gB gene (nt 703–1429 in EHV-2 accession number NC_001650.2 and nt 700–1426 in EHV-5 accession number NC_026421.1). The sequences used included Polish EHV-2 sequences (*n* = 57), Polish EHV-5 sequences (*n* = 2), and international sequences of EHV-2 (*n* = 26) and EHV-5 (*n* = 7) sourced from GenBank. The Polish sequences obtained in the current study are labelled PL_EHV2_ID number_ stud number. Accession numbers for sequences from GenBank are included in the description of each sequence. The evolutionary history was inferred by using the maximum likelihood method based on the Kimura 2-parameter model. The tree with the highest log likelihood (− 6036.44) is shown. The percentage of trees in which the associated taxa clustered together is shown next to the branches. The tree is drawn to scale, with branch lengths measured in the number of substitutions per site. Evolutionary analyses were conducted in MEGA5 [43]. The phylogenetic groups 1, 2, and 3 as defined by Sharp et al. [33] are shown on the right, with new groups labelled 4, 5, and 6. Clades within group 1 are labelled A, B, and C. Samples from the same stud are labelled with the rectangle of the same colour

Based on the phylogenetic tree (Fig. 4), Polish EHV-2 sequences clustered within four main branches. Most (*n* = 51) clustered with overseas sequences (including Australian reference strain EHV-2.86/67) within the “main” branch, defined as group 1 by Sharp et al. [33]. This was a large group that could be further divided into at least three clusters of closely related sequences containing 31 (cluster A), six (cluster B) and 14 (cluster C) Polish EHV-2 sequences (Fig. 4). Two Polish EHV-2 sequences (PL_EHV2_39_VIII and PL_EHV2_44_IX) clustered within group 2 as defined by Sharp et al. [33], which also contained another reference Australian strain EHV-2.141. The remaining Polish EHV-2 sequences formed two separate clusters containing just one (PL_EHV2_22_IV) or three (PL_EHV2_31_VI, PL_EHV2_32_VI and PL_EHV2_53_XII) sequences each. We have designated these as groups 4 and 5, respectively. Another group of EHV-2 sequences that appeared to be closer related to EHV-5 than to EHV-2 was designated as group 6 (Fig. 4). The two Polish EHV-5 sequences from stud IV appeared to be genetically different and clustered within two different groups (Fig. 4).

## Discussion

The high prevalence of EHV-2/5 infection among Polish horses is consistent with data reported from other countries, although the relative frequency of EHV-2/5 detection varied between studies with EHV-2 [44, 45] or EHV-5 [12, 46–50] being most common. These differences may reflect true differences in the epidemiology of EHV-2/5 in different horse populations, but may also be a reflection of the age structure of the horses sampled or the type of samples collected.

The viral load for both EHV-2 and EHV-5 was highest in swabs from young horses (foals and yearlings) for both EHV-2 and EHV-5. This was similar to findings reported by Hue et al. [42], but in contrast to those reported by Laabassi et al. [44], who did not find any differences between viral loads for horses of different ages. This discrepancy may be explained by the fact that in the latter study all horses 3-year-old and younger were considered together in one category, or may reflect other host- or virus-related differences between the populations studied. In the current study, the viral DNA load of EHV-2 was approximately 2 logs higher in nasal secretions from foals than from horses in all other age groups. It was also considerably higher than the levels of EHV-5 DNA shed by foals (Fig. 2). The reasons for this apparent high level of EHV-2 replication in upper respiratory tract of EHV-2 infected foals remain to be elucidated, but may explain efficient transmission of EHV-2 between foals and other in-contact animals.

While both EHV-2 and EHV-5 infections were common in young horses, EHV-2 infection was most common among foals, while EHV-5 infection was most common among yearlings, with foals being less likely to be infected with EHV-5 than older animals. Similar dynamics of EHV-2 and EHV-5 infections have been described recently among French horses [51], and EHV-5 infection has been reported to occur later than EHV-2 infection in studies were foals were followed for a period of time [9, 52, 53]. The reasons for these apparent differences in the epidemiology of two closely related equine γ-herpesviruses remain unclear. Based on considerably higher loads of EHV-2 compared to EHV-5 in nasal secretions of foals, one could speculate that EHV-2 may outcompete EHV-5 in dual infections of foals. In support of this hypothesis, EHV-2 was reported to grow faster than EHV-5 in vitro [26]. Whatever factors may facilitate efficient replication of EHV-2 in respiratory tract of foals, this apparent advantage seems to disappear with age, with similar levels of EHV-2 and EHV-5 shedding detected in nasal secretions from yearlings sampled in the current study.

Although foals and yearlings were most commonly positive for at least one virus, approximately 80% of 71 horses older than 10 years were also shedding EHV-2 or EHV-5. This was similar to a recent report from Algeria [44] where nasal swabs from 83.7% of 43 horses older than 10 years were positive for EHV-2, and 60.5% for EHV-5. However, all horses sampled by Laabassi and others [44] showed clinical signs of respiratory disease at the time of sampling. In contrast, in a recent Ethiopian study only 1/14 healthy and 1/9 diseased horses in the same age group were positive for EHV-2, with 2/9 diseased horses and none of healthy horses positive for EHV-5 [50]. In the current study, all older horses sampled comprised mares with foals at foot. It is possible that local immunosuppression associated with pregnancy [54] or stress related to birth induced recrudescence of latent EHV-2/5 infections in some mares, with subsequent horizontal spread to their foals, as has been suggested by others. Considering high frequency of EHV-2/5 shedding among foals, combined with high viral DNA load detected in nasal secretion of foals compared with older horses, it is likely that the presence of foals facilitated further spread of these viruses among in-contact animals, including other foals and mares, which resulted in the relatively high proportion of older horses positive for EHV-2/5 in the current study.

Similarly, the apparent seasonality of EHV-2 shedding (Table 3) was likely to be related to the seasonality of the breeding season and associated fluctuation in the age structure of horses present at studs, with most foals being born in spring.

Out of the breeds included in the study, Silesian and Polish Konik horses were less likely to be infected with either EHV-2 or EHV-5 than horses of other breeds. For Silesian horses, which represent a heavy type of Polish warmbloods, that may reflect the relatively older population sampled at stud X (median age of 4 years as opposed to 0.5 to 2 years for most other studs sampled). However, the age structure of the population of Polish Koniks sampled was similar to the age structure of horses at studs with other breeds. In addition, the virus load in nasal secretion of Polish Koniks was similar to the virus load secreted by horses of other breeds. Thus, factors other than the age structure or virus load must have influenced the comparatively limited spread of EHV-2/5 among Polish Koniks sampled.

We were unable to make any causative associations between EHV-2/5 infection and disease due to the fact that control healthy horses were not available for sampling on studs where diseased horses resided. In addition, as the samples were tested only for equine herpesviruses, we could not exclude the possibility that pathogens not tested for circulated among diseased horses and contributed to clinical disease observed, as suggested by results of a number of other studies [11, 55, 56]. Interestingly, there appeared to be differences in virus load in nasal secretions between diseased and healthy Arabian horses, which was particularly evident for EHV-2. This corresponds well with a higher blood EHV-2 DNA load in 2-month-old foals suspected of *Rhodococcus equi* pneumonia compared with healthy foals reported by others [57]. The reasons behind these findings, as well as the role of EHV-2 infection in development of respiratory disease needs further investigation.

Based on the phylogenetic analysis of a partial gB nucleotide sequence, Polish EHV-2 sequences clustered into at least four main groups, two of which were not originally described by Sharp et al. [33] based on the analysis of a comparable region that included hypervariable site III. This is not surprising considering a larger sample size used in the current study (83 versus 15 EHV-2 sequences), and highlights the high diversity of EHV-2 viruses. It is possible that the variability between Polish EHV-2 was even greater than reported, as DNA from 17 EHV-2 qPCR positive cultures did not yield expected gB products when tested with conventional primers, suggesting that there were nucleotide changes at the primer-binding sites that prevented binding of the primers used. Alternatively, lack of amplification of the conventional PCR product may reflect the quality of template DNA, as qPCR was designed to amplify a much shorter product (~ 78 bp) than conventional PCR (> 1300 bp). Altogether, our results agree with the results of others [9, 25, 47, 50, 58], and confirm high genetic variability between field EHV-2 viruses. The importance of this variability on biological properties of the virus and interactions with its equine host remains to be elucidated.

Interestingly, a group of four EHV-2 sequences including two sequences from Iceland [58] appeared to be closer related to EHV-5 than to EHV-2 based on the phylogenetic analysis, although the node separating these four EHV-2 sequences from EHV-5 sequences was positioned deep in the tree, with long branches separating the two groups. Another group of two EHV-2 viruses including Swe-2 was clearly separated from the remaining EHV-2 sequences. Based on a similar position of the Swe-2 sequence (group 3) on the phylogenetic tree constructed by Sharp et al. [33], the authors suggested that this virus may have arisen by recombination between EHV-2 and EHV-5. While this is one possible explanation that requires further investigation, it is also possible that viruses within group 3 and a newly designated group 4 represent EHV-2 viruses that diverged from other EHV-2 viruses early in the process of evolution.

It has been shown previously by several authors that foals can be hosts to more than one genotype of EHV-2 [25] and EHV-5 [27, 59]. Hence, the variability of EHV-2/5 among horses from the same stud (e.g. EHV-2 from stud IV, VII or stud IX and EHV-5 from stud IV) may represent ongoing introduction of viruses from outside sources, but may also simply indicate that a heterogenous population of EHV-2 viruses circulated among horses on those studs.

Interestingly, all four EHV-2 sequences from Polish Koniks were identical to each other, as were all three sequences from Silesian horses. Horses from these two breeds were significantly less likely to be infected with EHV-2 (and also EHV-5) than horses from other breeds. This observation is based on a very small number of samples, but the possibility that there is some as yet undefined relationship between breed and heterogeneity of EHV-2 warrants further investigation considering that similarly small number of samples from other studs/breeds yielded a more heterogenous population of EHV-2 sequences.

In the current study, 11/13 EHV-2 sequences obtained from horses with respiratory disease appeared to be closely related to each other based on the phylogenetic analysis. However, there was not a clear separation between these viruses and EHV-2 sequences from healthy horses, as evidenced by close similarity of EHV-2 sequences from sick and healthy Arabian horses from stud I. The possibility that genomic variability between EHV-2, and to lesser extent EHV-5, may explain somewhat poorly defended relationships between infection with these viruses and disease has been suggested by others, but, similarly to our results, there was no conclusive evidence that this may be the case [25].

## Conclusions

The results of the present study demonstrate that, similarly to other countries, equine γ-herpesvirus infections are common among Polish horses of various breeds and ages. The frequency of infection and viral load in nasal secretions were highest in young horses, which was particularly evident for EHV-2 infected foals and EHV-5 infected yearlings. Together with the relatively high frequency of EHV-2/5 infection among adult horses sampled, this suggests that the presence of foals and yearlings on a property is likely to increase the risk of active EHV-2/5 infection among in-contact horses. This is also a likely explanation for an increased frequency of EHV-2 infections in spring, when the highest numbers of foals are typically present at the breeding studs. While our data suggested for the first time the existence of breed-specific differences in susceptibility to EHV-2/5 infections, this would need to be further confirmed. If true, such differences may need to be considered in assessment of associations between EHV-2/5 infection and disease, which are currently poorly defined. The viral load of EHV-2 DNA was higher in diseased Arabian horses compared to healthy Arabian horses of the same age. This was unlikely to be attributed to the differences between EHV-2 viruses circulating among those horses. Despite a relatively high level of heterogeneity observed overall among the viruses examined, there was not a clear difference between a limited number of EHV-2 genotypes from diseased and healthy horses. Increased levels of EHV-2 shedding by horses affected by respiratory disease compared with healthy horses, particularly evident for EHV-2 infected Arabian foals, suggested increased levels of EHV-2 replication in diseased foals/horses. It remains to be seen whether this was directly linked to the development of disease or merely a result of a permissive environment in the respiratory tract created by some other, as yet undefined, factors (e.g. co-infection with other respiratory pathogens).

## Abbreviations

aa: Amino acid
ATCC: American Type Culture Collection
CPE: Cytopathic effect
Cq: Cycle quantitation
EHV-1: Equine herpesvirus 1
EHV-2: Equine herpesvirus 2
EHV-4: Equine herpesvirus 4
EHV-5: Equine herpesvirus 5
EHVs: Equid herpesviruses
gB: Glycoprotein B
ML: Maximum likelihood
qPCR: Quantitative polymerase chain reaction
RK-13: Rabbit kidney 13
